# Supplementary material for: Synthesis and Structural Characterization of a Silver(I) Pyrazolato Coordination Polymer
Source: Molecules. 2021 Feb 15;26(4):1015. doi: 10.3390/molecules26041015 (PMC7919055; doi:10.3390/molecules26041015)
Supplement: Supplementary file 1 [file molecules-26-01015-s001.pdf]

Supplementary Materials:

Synthesis and Structural Characterization of a Silver(I) Pyrazolato Coordination Polymer

Kiyoshi Fujisawa, Takuya Nemoto, Yui Morishima and Daniel B. Leznoff

Contents

**Figure S1**	1-D polynuclear structure of [Ag(μ-pz)]$_n$.	S-2
**Figure S2**	Packing diagram of [Ag(μ-pz)]$_n$.	S-2
**Figure S3**	Powder X-ray diffraction spectra of **[Ag(μ-L1Clpz)]$_n$** and calcd. pattern.	S-3
**Figure S4**	Packing diagram of **[Ag(μ-L1Clpz)]$_n$**.	S-4
**Figure S5**	Crystal structure of {[Ag(μ-L1Clpz)]$_3$}$_2$.	S-5
**Figure S6**	Packing diagram of {[Ag(μ-L1Clpz)]$_3$}$_2$.	S-5
**Figure S7**	^{1}H-NMR spectrum of **[Ag(μ-L1Clpz)]$_n$**.	S-6
**Table S1**	Comparisons of the ^{1}H-NMR chemical shifts.	S-6
**Figure S8**	UV-Vis spectra of **[Ag(μ-L1Clpz)]$_n$** and {[Ag(μ-L1Clpz)]$_3$}$_2$ in cyclohexane.	S-7
**Figure S9**	Luminescence spectra of **[Ag(μ-L1Clpz)]$_n$** and {[Ag(μ-L1Clpz)]$_3$}$_2$ in C_6H_{12}.	S-7
**Figure S10**	IR spectra of **[Ag(μ-L1Clpz)]$_n$** and {[Ag(μ-L1Clpz)]$_3$}$_2$	S-8
**Figure S11**	Raman spectra of **[Ag(μ-L1Clpz)]$_n$** and {[Ag(μ-L1Clpz)]$_3$}$_2$	S-8
**Figure S12**	Temperature dependent photoluminescence spectra in {[Ag(μ-L1Clpz)]$_3$}$_2$	S-9

References S-9

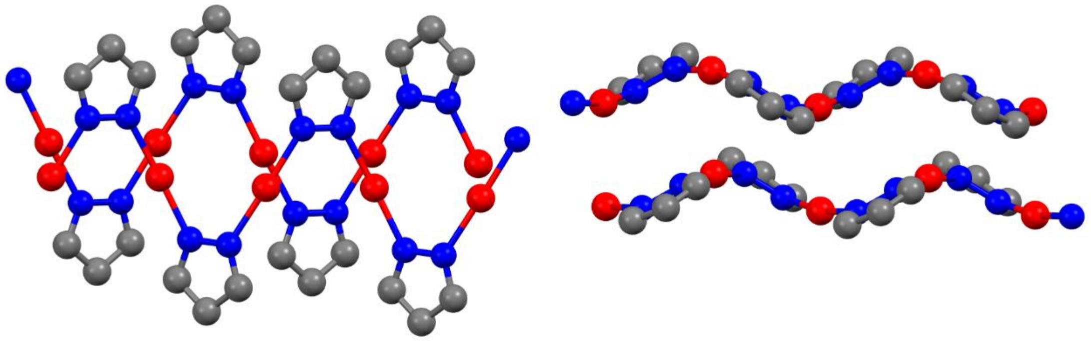

Figure S1. 1-D polynuclear structure of [Ag(μ-pz)]$_n$ (left) top view and (right) side view. Hydrogen atoms were omitted for clarity [1]. Color: red, silver; blue, nitrogen; grey, carbon. Intramolecular Ag···Ag, 3.3718(7) Å, intermolecular Ag···Ag 3.2547(6) Å.

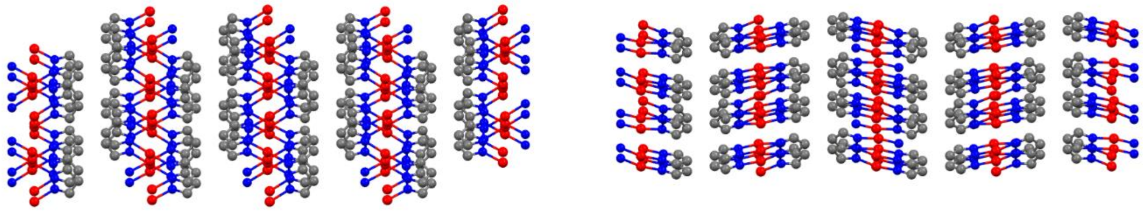

Figure S2. Packing diagram of [Ag(μ-pz)]$_n$ (left) top view and (right) side view. Hydrogen atoms were omitted for clarity [1]. Color: red, silver; blue, nitrogen; grey, carbon.

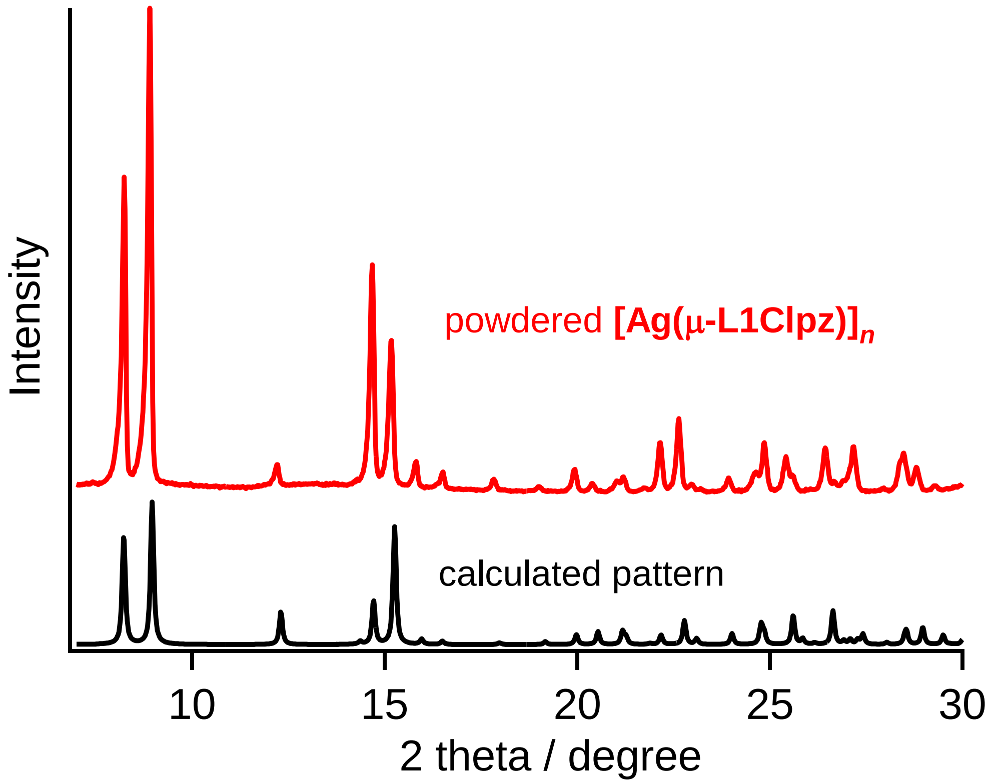

Figure S3. Powder X-ray diffraction spectrum of the obtained white powdered **[Ag(μ-L1Clpz)]*n*** (red line) and calculated X-ray diffraction pattern from single-crystal data (black line).

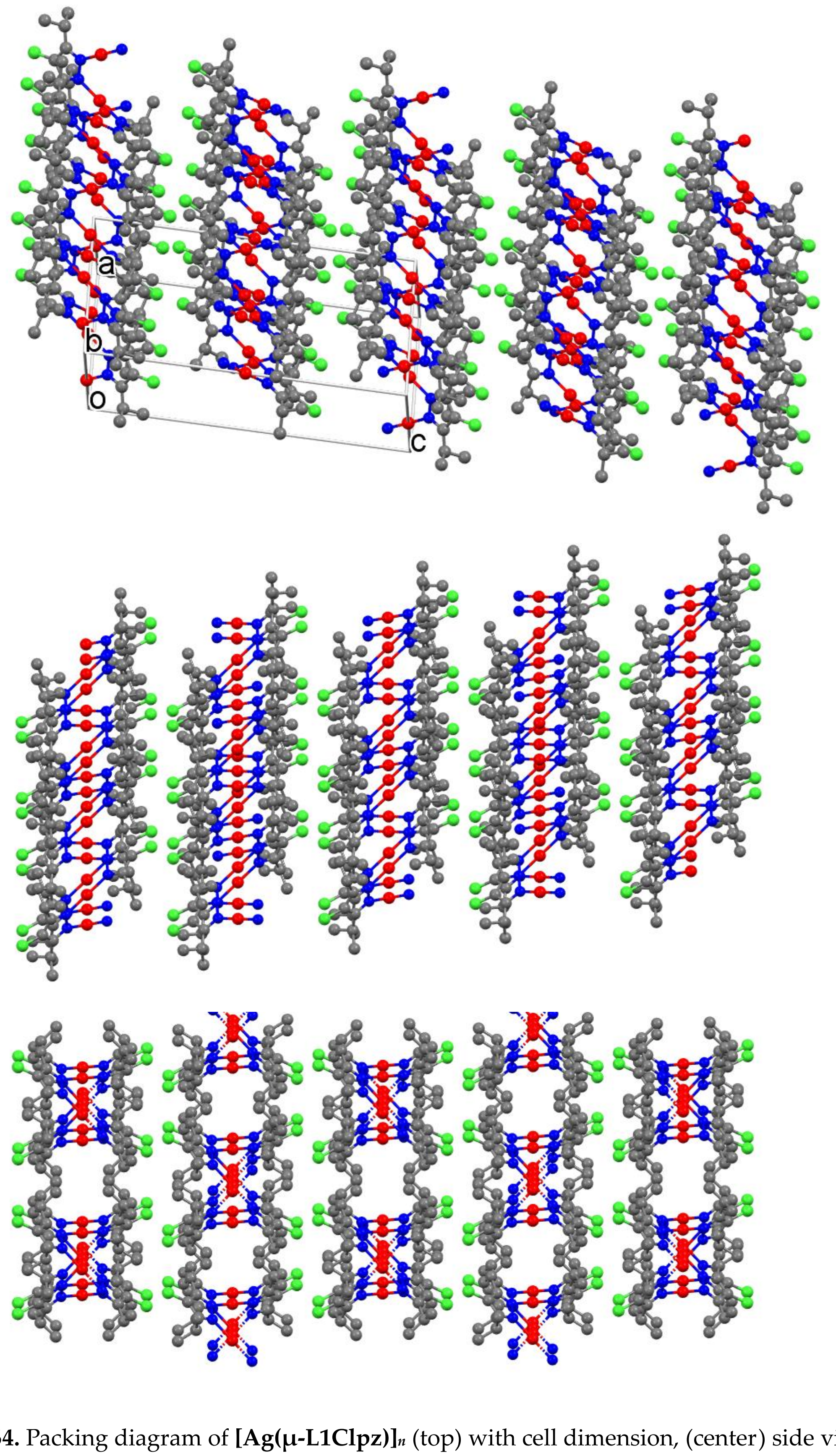

Figure S4. Packing diagram of **[Ag(μ-L1Clpz)]*n*** (top) with cell dimension, (center) side view, (bottom) top view. Hydrogen atoms were omitted for clarity. Color: red, silver; blue, nitrogen; green, chlorine; grey, carbon.

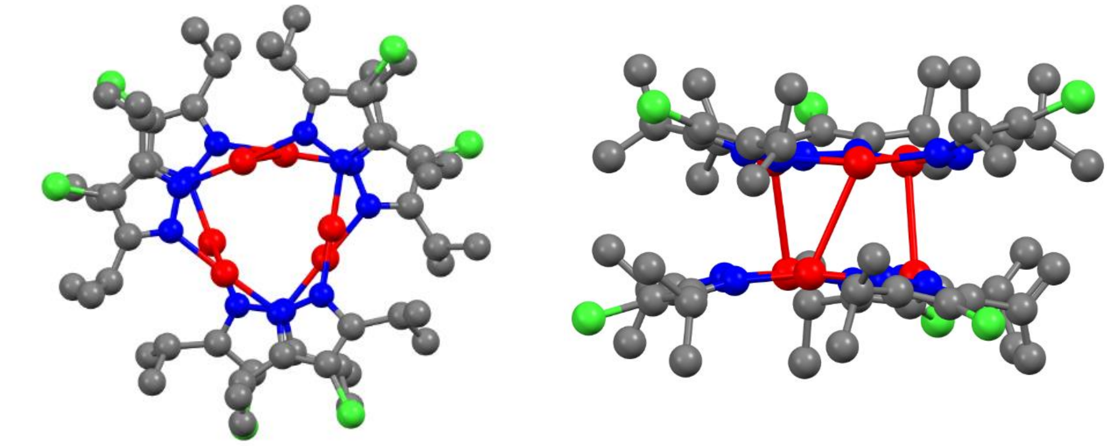

Figure S5. Crystal structure of $\{[Ag(\mu\text{-}L1Clpz)]_3\}_2$ (left) top view and (right) side view. Hydrogen atoms were omitted for clarity [2]. Color: red, silver; blue, nitrogen; green, chlorine; grey, carbon. Intramolecular Ag···Ag′ distances, 3.1003(17), 3.1298(15), and 3.1051(16) Å.

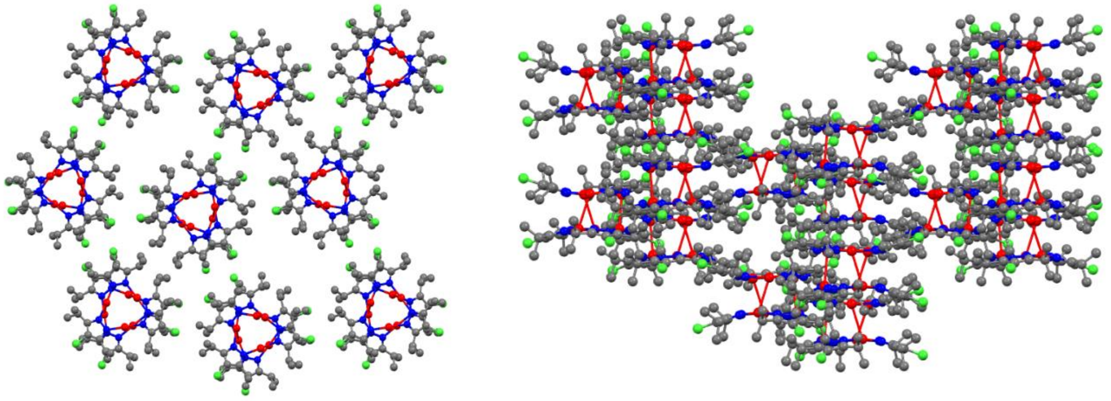

Figure S6. Packing diagram of $\{[Ag(\mu\text{-}L1Clpz)]_3\}_2$. (left) top view and (right) side view. Hydrogen atoms were omitted for clarity [2]. Color: red, silver; blue, nitrogen; green, chlorine; grey, carbon.

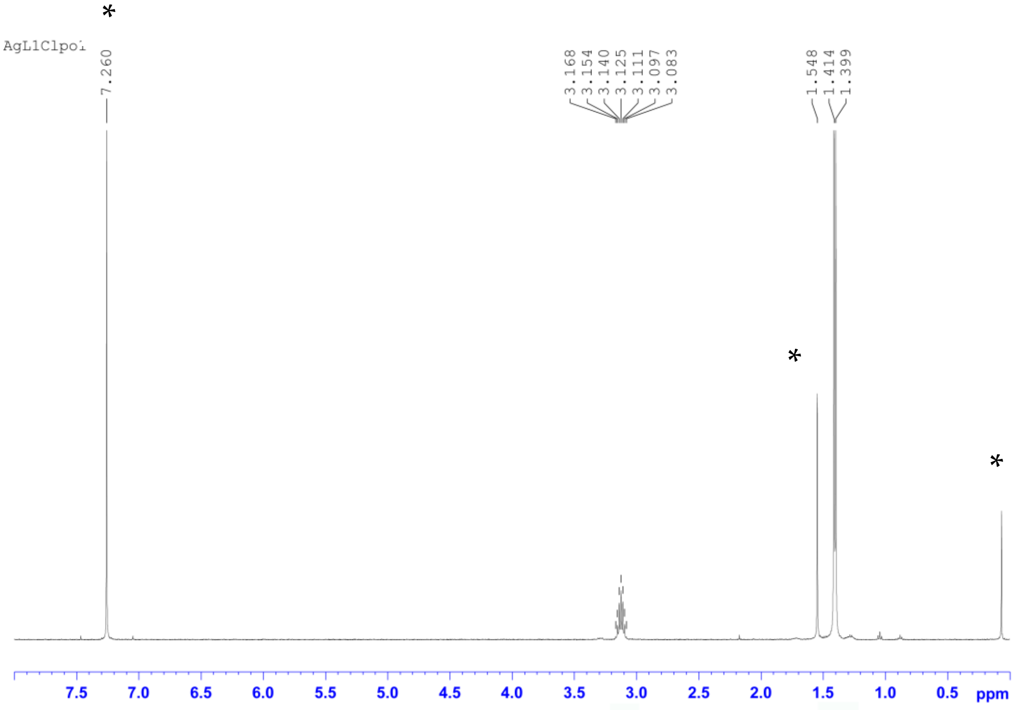

Figure S7. ^{1}H-NMR spectrum of **[Ag(μ-L1Clpz)]*n*** in $CDCl_3$ at room temperature (* marks solvents, water, and TMS peaks).

Table S1. Comparisons of the ^{1}H-NMR chemical shifts.

	**[Ag(μ-L1Clpz)]*n***	{[Ag(μ-L1Clpz)]$_3$}$_2$ [2]
CH*Me*$_2$	1.41	1.41
*CH*Me$_2$	3.13	3.13

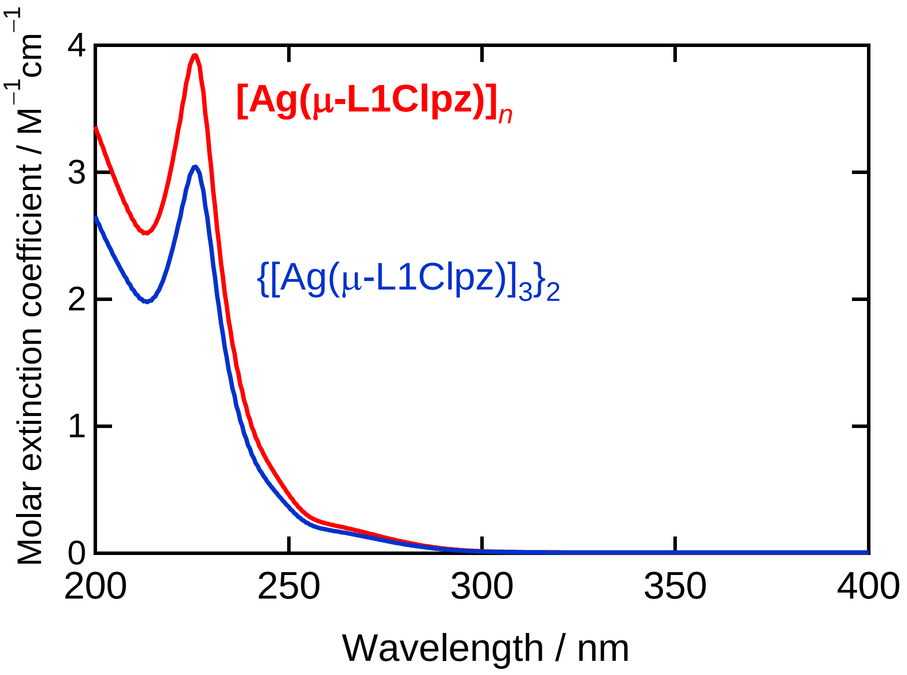

Figure S8. UV-Vis spectra of **[Ag(μ-L1Clpz)]*n*** (red line) and {[Ag(μ-L1Clpz)]$_3$}$_2$ (blue line) [2] in cyclohexane at room temperature.

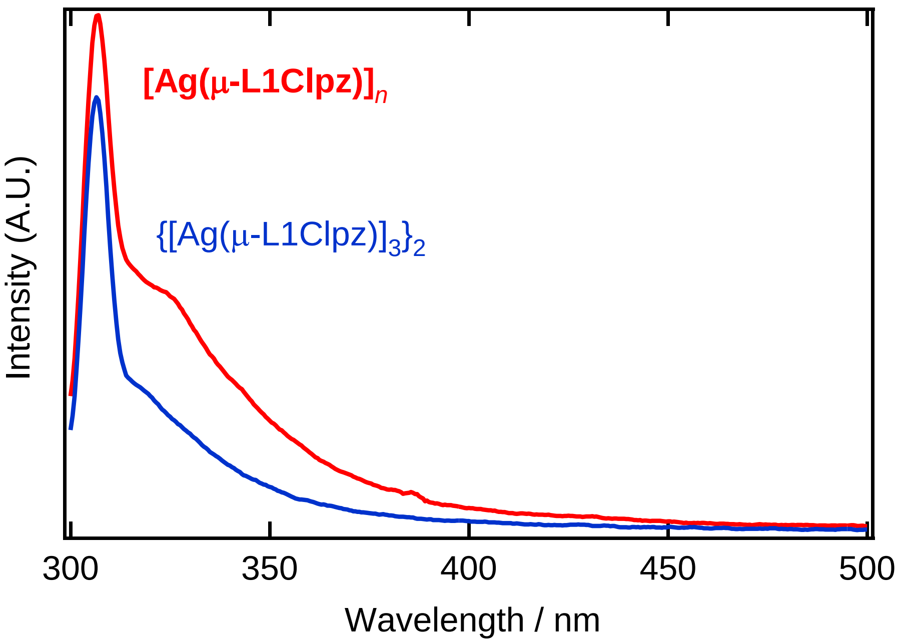

Figure S9. Photoluminescence spectra of **[Ag(μ-L1Clpz)]*n*** (red line) and {[Ag(μ-L1Clpz)]$_3$}$_2$ (blue line) [2] in cyclohexane at room temperature at 280 nm excitation wavelength.

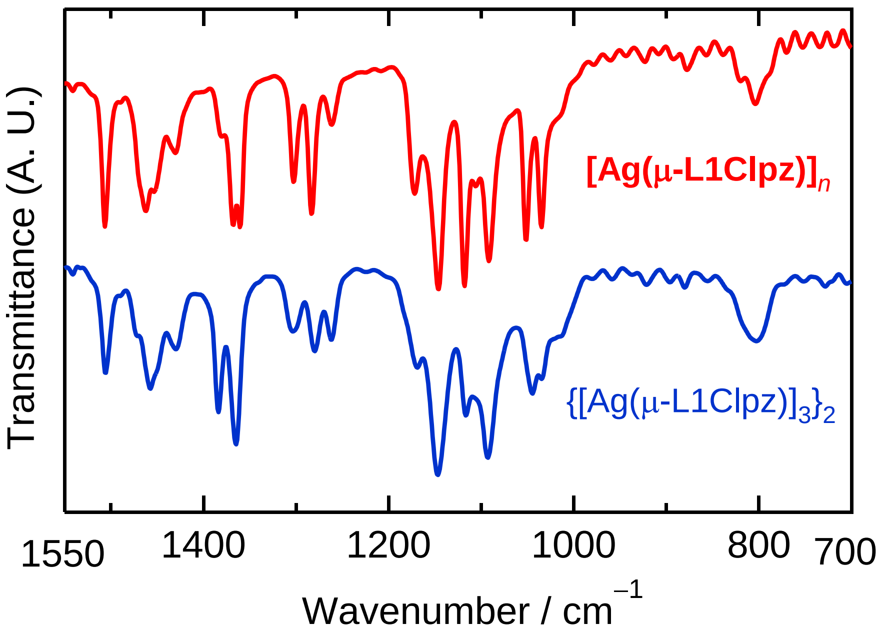

Figure S10. IR spectra of **[Ag(μ-L1Clpz)]*n*** (red line) and {[Ag(μ-L1Clpz)]3}2 (blue line) [2] in KBr disk at room temperature.

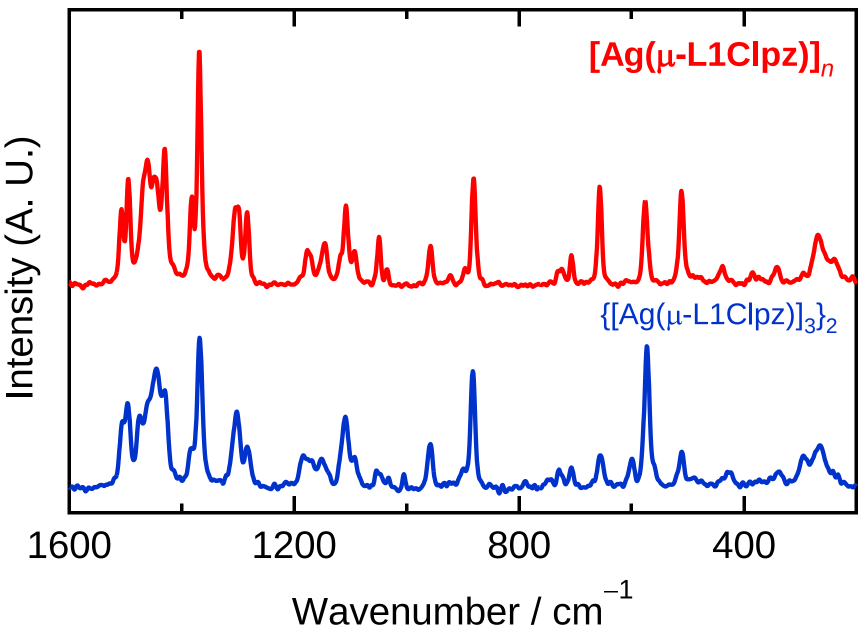

Figure S11. Raman spectra of **[Ag(μ-L1Clpz)]*n*** (red line) and {[Ag(μ-L1Clpz)]3}2 (blue line) [2] in powder at room temperature.

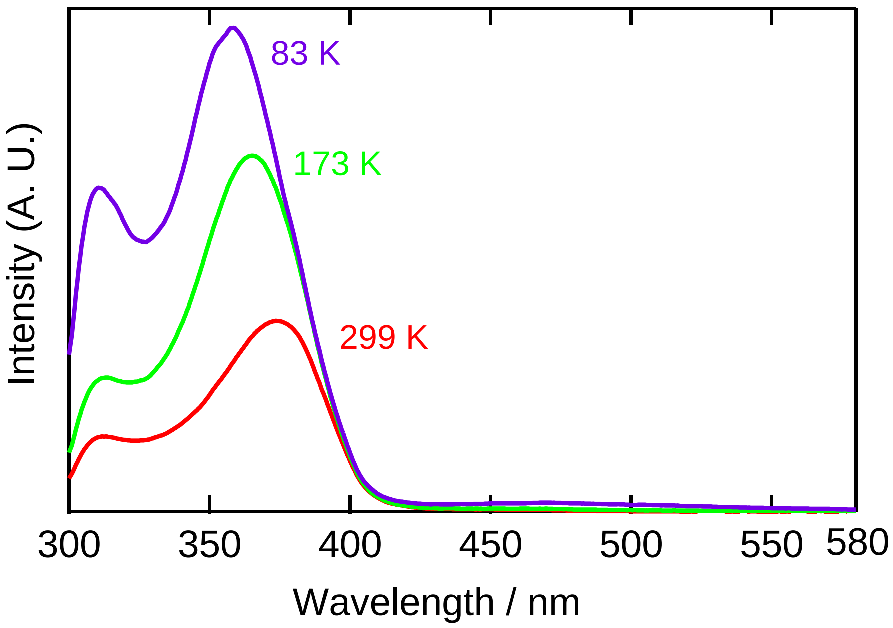

Figure S12. Solid-state temperature dependent photoluminescence spectra at 83 K (violet line), 173 K (green line), and 299 K (red line) in $\{[Ag(\mu\text{-L1Clpz})]_3\}_2$ at 280 nm excitation.

References

1. Zhang, C.-Y.; Feng, J.-B.; Gao, Q.; Xie, Y.-B. *catena*-Poly[silver(I)-*μ*-pyrazolato-$\kappa^2 N$:*N′*]. *Acta Cryst.* **2008**, *E64*, m352.
2. Morishima, Y.; Young, D. J.; Fujisawa, K. Structure and photoluminescence of silver(i) trinuclear halopyrazolato complexes. *Dalton Trans.* **2014**, *43*, 15915−15928.
